# Characterization of Gut Microbiota and Exploration of Potential Predictive Model for Hepatocellular Carcinoma Microvascular Invasion

**DOI:** 10.3389/fmed.2022.836369

**Published:** 2022-03-17

**Authors:** Ningning Zhang, Zeyu Wang, Jiayu Lv, Shuwen Zhang, Yang Liu, Tian Liu, Wang Li, Lan Gong, Xiaodong Zhang, Emad M. El-Omar, Wei Lu

**Affiliations:** ^1^Department of Hepatobiliary Oncology, Liver Cancer Center, Tianjin Medical University Cancer Institute and Hospital, National Clinical Research Center for Cancer, Key Laboratory of Cancer Prevention and Therapy, Tianjin's Clinical Research Center for Cancer, Tianjin Medical University, Tianjin, China; ^2^Tianjin's Clinical Research Center for Cancer, Tianjin Medical University, Tianjin, China; ^3^Department of Hepatology, Tianjin Third Central Hospital, Tianjin, China; ^4^Department of Medicine, University of New South Wales, Sydney, NSW, Australia; ^5^St George & Sutherland Clinical School, Microbiome Research Centre, University of New South Wales, Sydney, NSW, Australia; ^6^Key Laboratory of Cancer Prevention and Therapy, Department of Gastrointestinal Cancer Biology, Liver Cancer Center, National Clinical Research Center for Cancer, Tianjin Medical University Cancer Institute and Hospital, Tianjin Medical University, Tianjin, China

**Keywords:** hepatocellular carcinoma, microvascular invasion, gut microbiota, predictive model, tumor microenvironment, M2-type TAM, mTOR signaling pathway

## Abstract

**Background:**

The association between gut microbiota and microvascular invasion (MVI) in patients with hepatocellular carcinoma (HCC) remains unclarified. Hence, the microbiome analysis of patients with HCC might predict MVI development as an accurate, non-invasive, and convenient assessment. The aim of this study was to investigate the characteristics of gut microbiota in patients with HCC-MVI and establish a microbial prediction model of HCC-MVI based on a microbiome study.

**Methods:**

Fecal samples were collected from 59 patients with HCC (24 of the total with MVI disease and 16 healthy controls) and were further analyzed by 16S rRNA amplicon sequencing followed by a comprehensive bioinformatic analysis. The diagnostic performance of microbiome characteristics in predicting MVI was assessed by receiver operating characteristic (ROC) curves. The correlation between gut microbiota and tumor microenvironment (TME) in the HCC-MVI group was further analyzed by using immunohistochemistry and immunofluorescence assay.

**Results:**

A significant differentiation trend of microbiota composition and structure was observed between the HCC-MVI group and those without vascular invasion (HCC-NVI). Compared with HCC-NVI group and healthy controls, gut bacteria *Klebsiella, Proteobacteria, Prevotellaceae*, and *Enterobacteriaceae* were significantly enriched, whereas *Firmicutes, Ruminococcus*, and *Monoglobaceae* were significantly decreased in patients with HCC-MVI. *Klebsiella* was considered to be the key microbiome signature for patients with HCC-MVI. The area under the curve (AUC) of the established HCC-MVI microbial prediction model was 94.81% (95% CI: 87.63–100%). The percentage of M2-type tumor-associated macrophages (TAMs) was increased in the HCC-MVI group compared with the HCC-NVI group (*p* < 0.001). M2-type TAMs in TME were negatively correlated with Shannon and Simpson index of HCC-MVI gut microbiota (all *p* < 0.01). In addition, predicted KEGG pathways showed that the functional differences in the metabolic pathways of microbiota varied among the groups.

**Conclusion:**

The results indicated that differences existed in the fecal microbiome of patients with HCC-MVI and healthy controls. The prediction model of HCC-MVI established with certain gut bacterial signatures may have the potential to predict HCC-MVI outcome, and the characteristics of the fecal microbiome in patients with HCC may be associated with TME, though future larger-cohort studies are required to validate this supposition.

## Introduction

Liver cancer is considered to be the fifth leading cause of cancer-related death (1, 2). The risk of recurrence after resection is up to 70% after 5 years, and the 5-year survival rate is as low as 18% (3). Hepatocellular carcinoma (HCC) is the most prevalent form of liver cancer (4), and most patients with HCC have metastatic disease or vascular invasion at their initial diagnosis (5–7). HCC tumors are heterogeneous, and patients at the same disease stage may have very different treatment outcomes (1).

Vascular invasion, including microvascular invasion (MVI), is one of the most critical factors in determining long-term survival and tumor recurrence for patients with HCC (8, 9). MVI is defined as a histological feature that is based on postoperative pathology (10–14). Once MVI develops, the tumor invades the blood vessels, and the current postoperative diagnosis of MVI leaves not much time window for therapeutic intervention. Accurate preoperative tests to predict MVI are thus urgently needed to reduce recurrence and improve the survival of patients with HCC. However, no such related methods, especially in a non-invasive manner, established to date have adequate sensitivity, specificity, or reproducibility for this purpose (15).

More than 100 trillion gut microorganisms form a complex microbial community, namely microbiota, that has a major impact on human health (16). Accumulating evidence has suggested a link between the microbiome, the metagenome of microbiota, and liver diseases. It is estimated that gut microbiota could be associated with 15–20% of patients with HCC (17). The gut–liver axis refers to the bidirectional relationship between the gut with its microbiota and the liver, which enables the transport of gut-derived products including microbial metabolites directly to the liver, and the liver feedback route of bile and antibody secretion to the intestine (18, 19). Previously, interactions between the intestinal microbiome and liver diseases were also reported in several studies, which showed the predictive value of gut microbiome in tumor prognosis, the effect on chemotherapy, and immunotherapy responses (20–22). Gut microbiota can regulate corresponding miRNAs through impacting circRNA expression and play a role in microbiota-mediated cancer metastasis (23). Li et al. identified CTSK as a mediator between the dysbiosis of gut microbiota and CRC metastasis (24). Gut or mammary duct colonization with enterotoxigenic bacteria *Bacteroides fragilis* triggers epithelial hyperplasia and augments breast cancer growth and metastasis (25).

The underlying mechanism of MVI in patients with HCC which induces metastasis and recurrence is very complicated and remains largely unclear (26). To the best of our knowledge, the correlation between gut microbiota and HCC-MVI remains uninvestigated, despite numerous reports linking gut dysbiosis with HCC pathogenesis. We hereby hypothesized that gut microbiota has a potential influence on HCC-MVI development, which may give credit to its role in cancer metastasis. Therefore, in this clinical study, we wanted to further investigate the relationship between the gut microbiota and HCC-MVI to gain a better understanding of the underlying interaction. To validate the previous research and provide deeper insights into the mechanism of fecal microbiome affecting HCC patients' MVI development, the current study was intended to further apply 16S rRNA amplicon sequencing and microbiome analysis to investigate the differences in taxonomic and derived functional profiles in the fecal microbiota from patients with HCC and healthy controls, clarify the predictive role of a key microbial signature in HCC-MVI, and further demonstrate its relevance to host immunity.

## Methods

### Subject Recruitment and Sample Collection

In this study, we identified 123 patients between January 2020 and December 2020 who had an initial presentation of hepatitis B virus (HBV)-related HCC at the Department of Liver Cancer Center, Tianjin Medical University Cancer Institute and Hospital. HBV-related HCC was defined here as seropositive for hepatitis B surface antigen (HBsAg), non-hepatitis C, and non-alcohol-related liver disease. The initial diagnosis of primary HCC is determined by pathology. Macrovascular invasion (MaVI) diagnosis is based on imaging (CT/MRI). The diagnosis of MVI is based on the postoperative pathological diagnosis. Patients with HCC without vascular invasion are grouped as HCC-NVI. Gender- and age-matched healthy volunteers with no history of diseases were enroll (1) concomitant with other malignant tumors; (2) nonnative patients who have received antitumor therapy; (3) combined with chronic diseases such as hypertension, diabetes, and gastrointestinal diseases; (4) use or take antibiotics, proton pump inhibitors, probiotic products 1 month before sampling; (5) missing clinical parameters. This study was approved by the Medical Ethics Committee at the Tianjin Cancer Hospital and written informed consent was obtained from all participants.

### DNA Isolation From Fecal Samples and 16S rRNA Amplicon Sequencing

Fecal specimens (10 ml) were thawed and centrifuged at 7,500 *g*, 4°C for 10 min. Total genomic DNA was extracted by DNA Extraction Kit (Tiangen, China) from fecal samples. The 16S rRNA genes of distinct regions (16S V3-V4) were amplified using specific primers with the barcode. The PCR reactions were performed by 0.2 μM of forward and reverse primers, 15 μl of Phusion® High-Fidelity PCR Master Mix (New England Biolabs, USA), and 10 ng of template DNA. PCR cycling was carried out by the following program: 98°C 1 min, 25 cycles of 98°C 15 s, 55°C 30 s, 72°C 30 s, and 72°C 5 min. After the PCR process, the products were detected by operating electrophoresis on a 2% agarose gel. Then, Qiagen Gel Extraction Kit (Qiagen, Germany) was used to purify the mixture PCR products. Sequencing libraries were produced by TruSeq® DNA PCR-Free Sample Preparation Kit (Illumina, USA) following the manufacturer's recommendations, and the index codes were added. At last, the library was sequenced on an Illumina NovaSeq platform and 250 bp paired-end reads were generated.

### Immunohistochemical Staining

The HCC tissues were serially sectioned at a thickness of 3–5 mm. Then, the tissues were dewaxed, deparaffinized in xylene, and rehydrated. The samples were then boiled for 15 min in a microwave oven for the purpose of increasing antigen retrieval. Then, 3% hydrogen peroxidase was used to block the endogenous peroxidases for 30 min. The slides were incubated with primary antibody CD8^+^ (PeproTech, 67786-1, 1:8000) overnight. Biotin-free horseradish peroxidase-labeled polymer of the Envision Plus detection system (Dako, Denmark) was used in the process of detection. Double immunofluorescence staining was performed as previously described. Paraffin-embedded sections were incubated with the following primary antibodies: F4/80 (Santa Cruz Biotechnology, SC-365340, 1:500), CD206 (PeproTech, 18704-1-AP, 1:800), and CD86 (CST, 91882S, 1:100) at 4°C overnight. Then, the sections were incubated at 37°C with the matched fluorescently labeled secondary antibodies (1:500; Invitrogen; CA, USA) for 30 min.

### Bioinformatics and Statistical Analyses

According to the QIIME (V1.9.1) quality control process, quality filtering of raw tags was conducted under specific filtering conditions for the purpose of obtaining the high-quality clean tag. Uparse software (Uparse v7.0.1001) was used for the analysis of the sequences. Similar sequences higher than 97% were defined by the same operational taxonomic units (OTUs). Silva Database (http://www.arb-silva.de/) based on the Mothur algorithm was applied to annotate taxonomic information of each representative sequence. All the indices were calculated with QIIME and displayed with R Software (Version 2.15.3). Cluster analysis was preceded by principal component analysis (PCA), which was used to reduce the dimension of the original variables by FactoMineR package and ggplot2 package. Principal coordinate analysis (PCoA) was displayed to get principal coordinates and visualized with the WGCNA package, stat packages, and ggplot2 package. To determine the enrichment in the assigned taxonomic and functional profiles, LEfSe analysis was performed. Taxonomic levels with LEfSe values higher than 4 at a *p* < 0.05 were statistically significant. Operating characteristic curves (receiving operational curve, ROC) were constructed, and then the area under the curve (AUC) was calculated to evaluate the discriminatory ability of the random forest model. General statistical analysis was performed in Prism v8.2.1 (GraphPad Software, Inc.), and one-way ANOVA with Tukey's multiple comparisons test was used to determine differences between the groups. Spearman's rank correlation was performed in R (v3.6.1) to analyze the relationships between the microbiome and disease status.

All *p*-values were corrected using Benjamini–Hochberg multiple test correction. *p-*values of < 0.05 were considered statistically significant.

## Results

### Baseline Clinical Characteristics of the Participants

**Figure 1:**
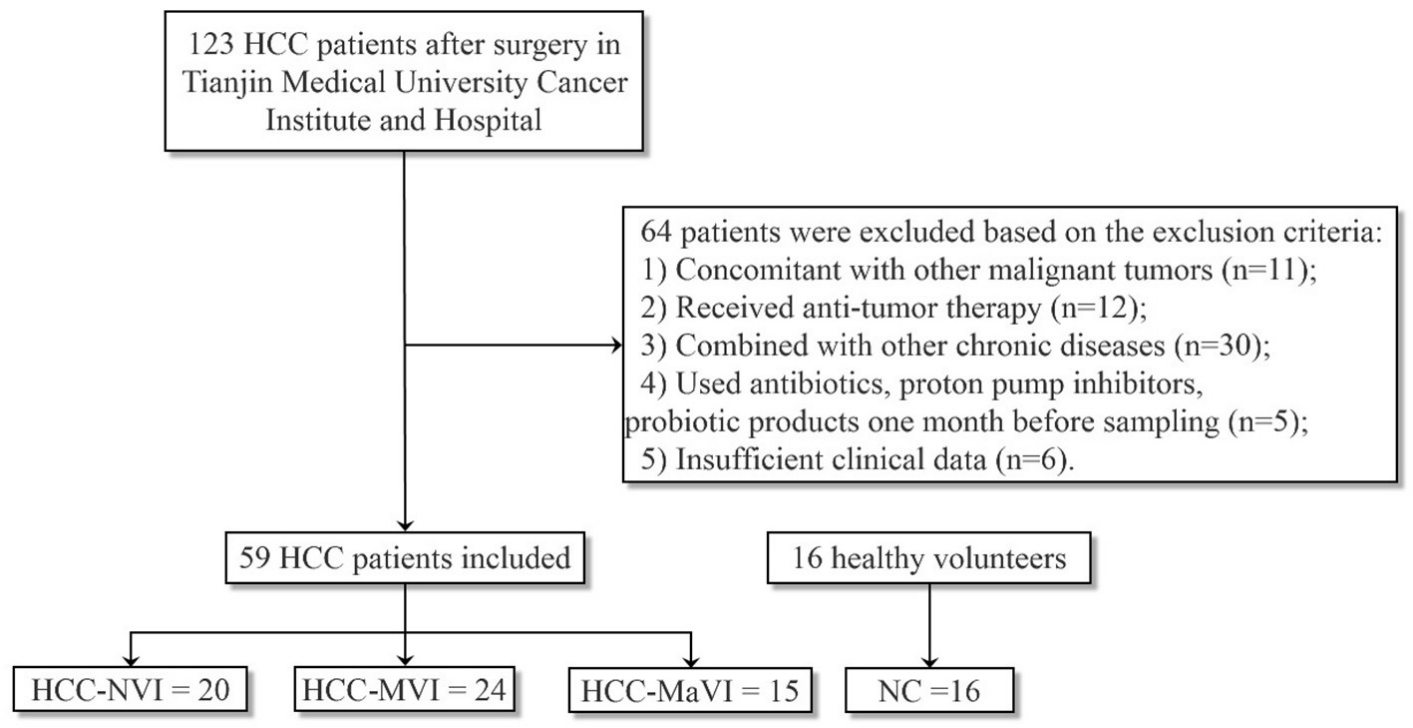
Flow diagram describing the selection of the study population.

Fecal samples were collected from a total of 75 subjects including 16 healthy controls (NC), 20 patients with HCC-NVI, 24 patients with HCC-MVI, and 15 patients with HCC-MaVI (Figure 1). The demographic and clinical data of the four groups of participants including 59 patients with HCC and 16 healthy controls were analyzed and displayed in Table 1. Based on the published clinical studies employing microbiome analyses of four or more groups, we performed sample size calculation using G^*^power software and estimated that at least 15 participants in each group would provide an estimated effect size of 0.405, which is adequate to detect clinically meaningful differences between the subgroups with a power of at least 80% with a two-tailed *p*-value of < 0.05. Comparisons of all four groups and three HCC groups demonstrated significant differences among the levels of total bilirubin (TBIL), which is regarded as an indicator for liver dysfunction (Supplementary Figure 1A) (1). Although there was no significant difference in alpha-fetoprotein (AFP) level among the three HCC groups, a statistically significant difference in AFP level among all the four groups was observed when healthy controls were taken into consideration (Supplementary Figure 1B). No significant difference in age, gender, BMI, and other available clinical parameters was elicited among these four groups.

**Table 1:** Clinical characteristics of all participants.

	**NC**	**HCC-NVI**	**HCC-MVI**	**HCC-MaVI**	***P-value***
	***n* = 16**	***n* = 20**	***n* = 24**	***n* = 15**	
Gender (Male)	10 (62.5%)	12 (60.0%)	20 (83.3%)	12 (80.0%)	0.252
Age, years	53.0 (8.0)	57.4 (8.6)	59.8 (12.1)	53.3 (11.4)	0.131
BMI, kg/cm^2^	24.4 (2.6)	24.6 (3.1)	24.4 (3.7)	24.8 (2.8)	0.980
Cirrhosis (yes)	-	13 (65.0%)	16 (66.7%)	12 (80.0%)	0.588
Etiology (HBV)	-	20 (100.0%)	24 (100.0%)	15 (100.0%)	NA
ALB, g/L	43.8 (2.0)	43.3 (3.8)	42.3 (4.6)	41.8 (6.6)	0.559
TBIL, mmol/L	14.4 (4.5)	15.0 (5.8)	15.7 (5.0)	20.5 (6.7)	0.011
AFP, ng/ml	9.23 (4.1)	4,957 (14,905.0)	6,807 (19,171.0)	40,324 (89,308.0)	0.035
Child-Pugh class (A/B)	-	20 (100.0%)/ 0 (0.0%)	24 (100.0%)/0 (0.0%)	13 (86.7%)/ 2 (13.3%)	0.061
AST (U/L)	32.0 (20.3)	32.5 (19.5)	35.6 (28.8)	41.0 (21.7)	0.685
PLT (10^9^/L)	197.56 (45.14)	185.00 (86.70)	173.83 (69.86)	195.33 (76.53)	0.717
Tumor largest size (cm)	NA	5.58 (3.23)	6.24 (4.06)	7.51 (2.87)	0.279

*AFP, alpha-fetoprotein; ALB, albumin; AST, aspartate aminotransferase; BMI, body mass index; HBV, hepatitis B virus; TBIL total bilirubin. Quantitative value was presented as Mean (SD)*.

### Alpha and Beta Diversities of Fecal Microbiome

**Figure 2:**
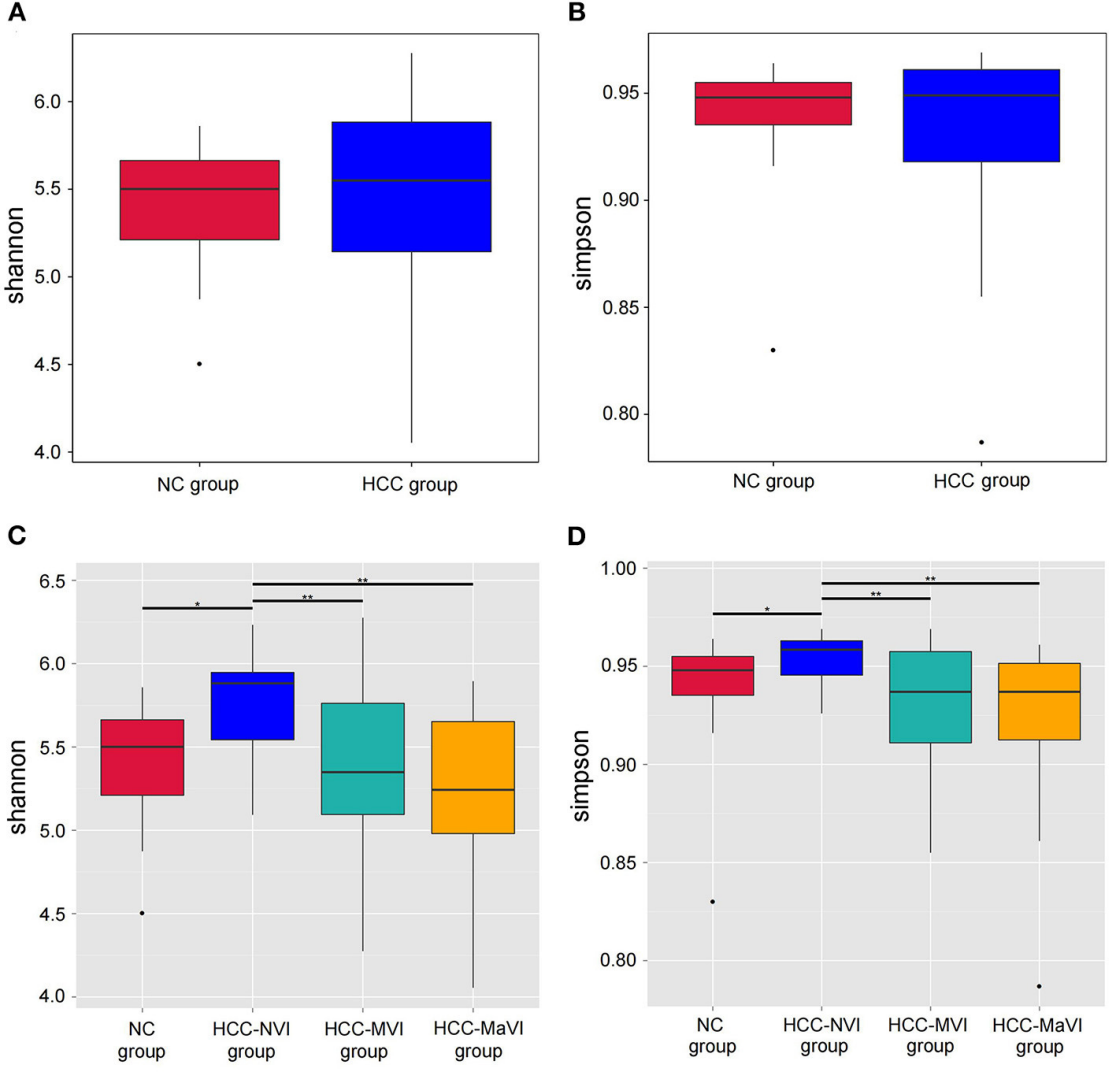
Comparison of alpha-diversity of the gut microbiota between patients with HCC and the NC group. Shannon **(A)** and Simpson **(B)** indexes were used to evaluate the evenness and richness in the HCC and NC groups; Shannon **(C)** and Simpson **(D)** indexes were used for comparing the evenness and richness in the NC, HCC-NVI, HCC-MVI, and HCC-MaVI groups. Difference in microbiota composition between the groups based on Bray-Curtis metrics was displayed by box-plot according to the Wilcoxon rank-sum test, *P < 0.05, **P < 0.01.

Richness and evenness analysis was performed in the fecal microbiome of the patients with HCC and healthy controls (NC) using the Shannon and Simpson index to display the alpha diversity (Figures 2A,B). No significant difference was detected. When compared with the NC or HCC-NVI group, the microbial alpha diversity within the HCC-VI (HCC-MVI or HCC-MaVI) group showed significant statistical differences (Figures 2C,D). However, the beta diversity in the patients with HCC and NC group exhibited significant differences under the multiple response permutation procedure (MRPP; A = 0.0291, *P* = 0.001). The result of MRPP in NC, HCC-MVI, and HCC-MaVI groups all showed significant differences (all A > 0, *P* < 0.001).

**Figure 3:**
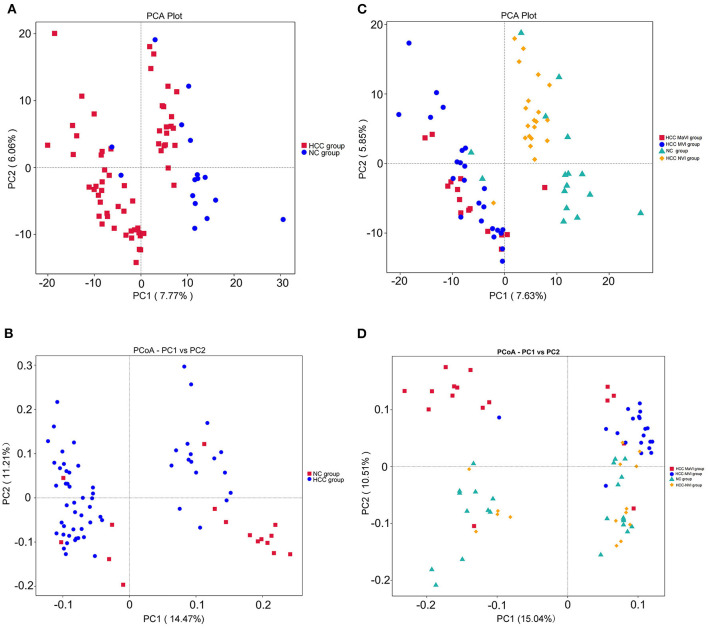
Comparison of beta diversity by principal component analysis (PCA) and principal coordinate analysis (PCoA) plots. PCA **(A)** and PCoA analysis of variation based on unweighted UniFrac distances **(B)** of NC and HCC groups. Red and blue dots represent HCC and normal controls, respectively. Each point represents an individual sample. PCA **(C)** and PCoA analysis of variation based on unweighted UniFrac distances **(D)** of NC, HCC-NVI, HCC-MVI, and HCC-MaVI groups. Red dots represent HCC-MaVI, blue dots represent HCC-MVI, yellow dots represent HCC-NVI, and green dots represent the NC group, respectively. Each point represents an individual sample.

The PCA and unweighted Unifrac principal coordinate analysis (PCoA) analysis indicated that the NC and HCC groups had no clustering with members of their own group (Figures 3A,B). Subgroup analysis demonstrated that no identified microbiota clusters were observed in NC, HCC-NVI, HCC-MVI, and HCC-MaVI groups with PCA and unweighted Unifrac PCoA analysis (Figures 3C,D).

### Taxonomic Profiles of the Gut Microbiome

**Figure 4:**
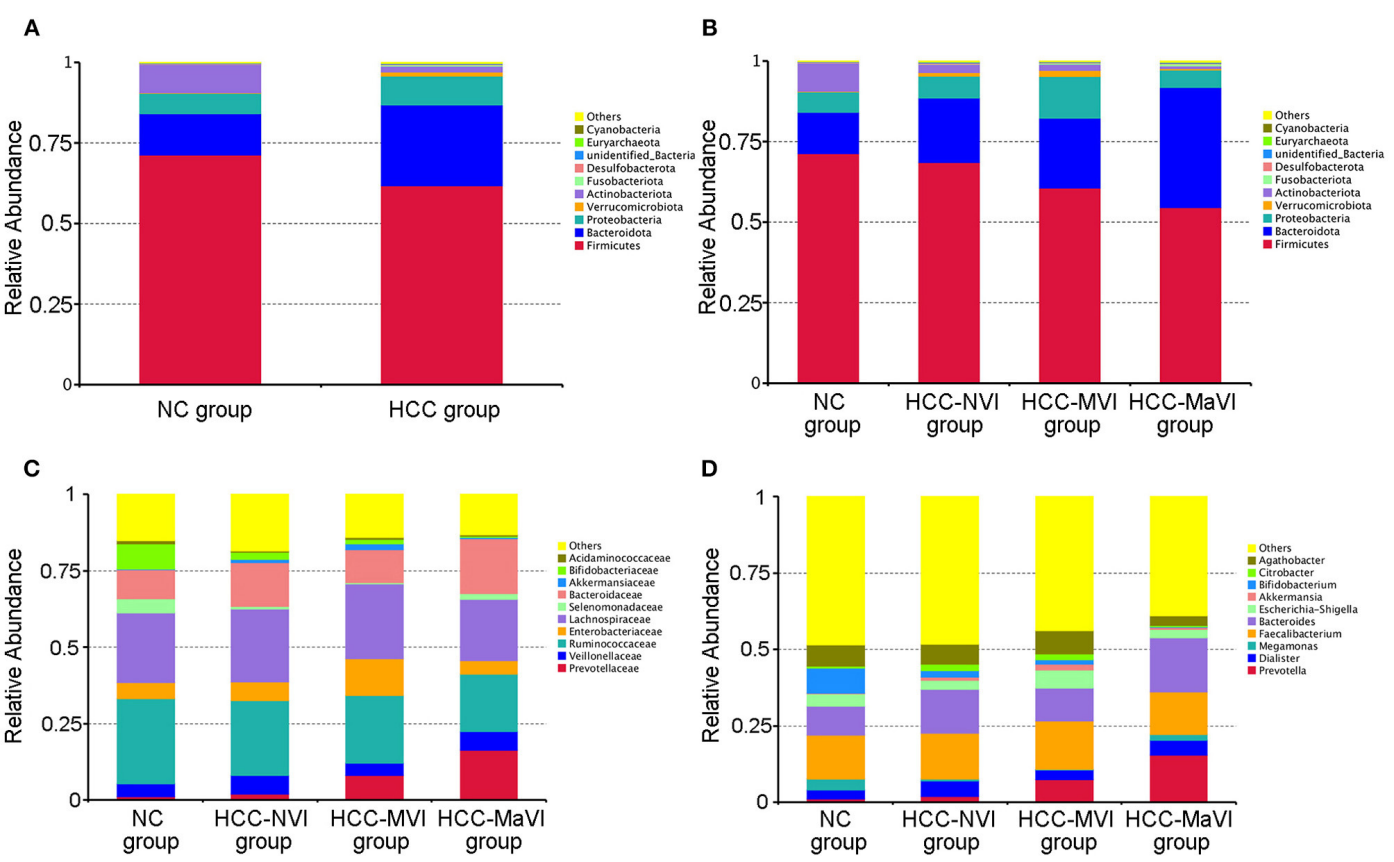
Relative abundance of microbiome across different groups. **(A)** The heatmaps of the NC and HCC bacteria groups at the phylum level. Heatmaps of the NC, HCC-NVI, HCC-MVI, and HCC-MaVI bacteria groups at the phylum level **(B)**, at the family level **(C)**, at the genus level **(D)**.

According to the relative abundance of the microbiota in the NC group, HCC samples, and their subgroups, classification and analysis were carried out at the levels of phylum, class, order, family, and genus. At the phylum level, the relative abundance of Firmicutes and Actinomycetes in the NC group was significantly greater than that of the HCC group (*P* = 0.004; 0.002), while the Bacteroidetes was significantly enriched in patients with HCC (*P* < 0.001; Figure 4A and Supplementary Figure 2). Firmicutes showed a significant decrease in the HCC-VI group when compared with the HCC-NVI group and the NC group. The relative abundance of Proteobacteria in the HCC-MVI group was greater than that of the HCC-NVI group and the NC group; the relative abundance of Bacteroides in the HCC-MaVI group was higher than that of the HCC-NVI group and the NC group, while the relative abundance of *Actinobacteria* was lower (Figure 4B and Supplementary Figure 3).

Under the phylum level, the abundance of *Bifidobacterium* was decreased in patients with HCC (P = 0.002), while *Prevotella* and *Klebsiella* were increased compared with the NC group. Compared with the HCC-NVI group and the NC group, the abundance of *Prevotellaceae* and *Enterobacteriaceae* was significantly increased in the HCC-MVI group while that of *Ruminococcaceae* was decreased. The abundance of *Ruminococcaceae, Bifidobacteriaceae*, and *Veillonellaceae* was significantly decreased in the HCC-MaVI group while *Bacteroidaceae* showed a substantial increase (Figure 4C). Compared with the HCC-NVI group and the NC group, the abundance of *Prevotella* and *Klebsiella* was significantly increased in the HCC-MVI group. The abundance of *Bifidobacterium* significantly decreased and *Prevotella* significantly increased in the HCC-MaVI group (Figure 4D). Differential abundance of microbes at the class, order, family, and genus levels are displayed in Supplementary Figures 4–10. Briefly, *Clostridia* and *Bacterodia* exhibited higher abundance in HCC groups at the class level, *Bacteroidales* was significantly increased in HCC groups at the order level, the abundance of *Prevotellaceae* in HCC groups was significantly increased while *Bifidobacteriaceae* was significantly decreased at the family level, and *Faecalibacterium* showed the highest abundance in either NC group or HCC groups at the genus level.

### Predicted Functional Profiles of the Gut Microbiome

**Figure 5:**
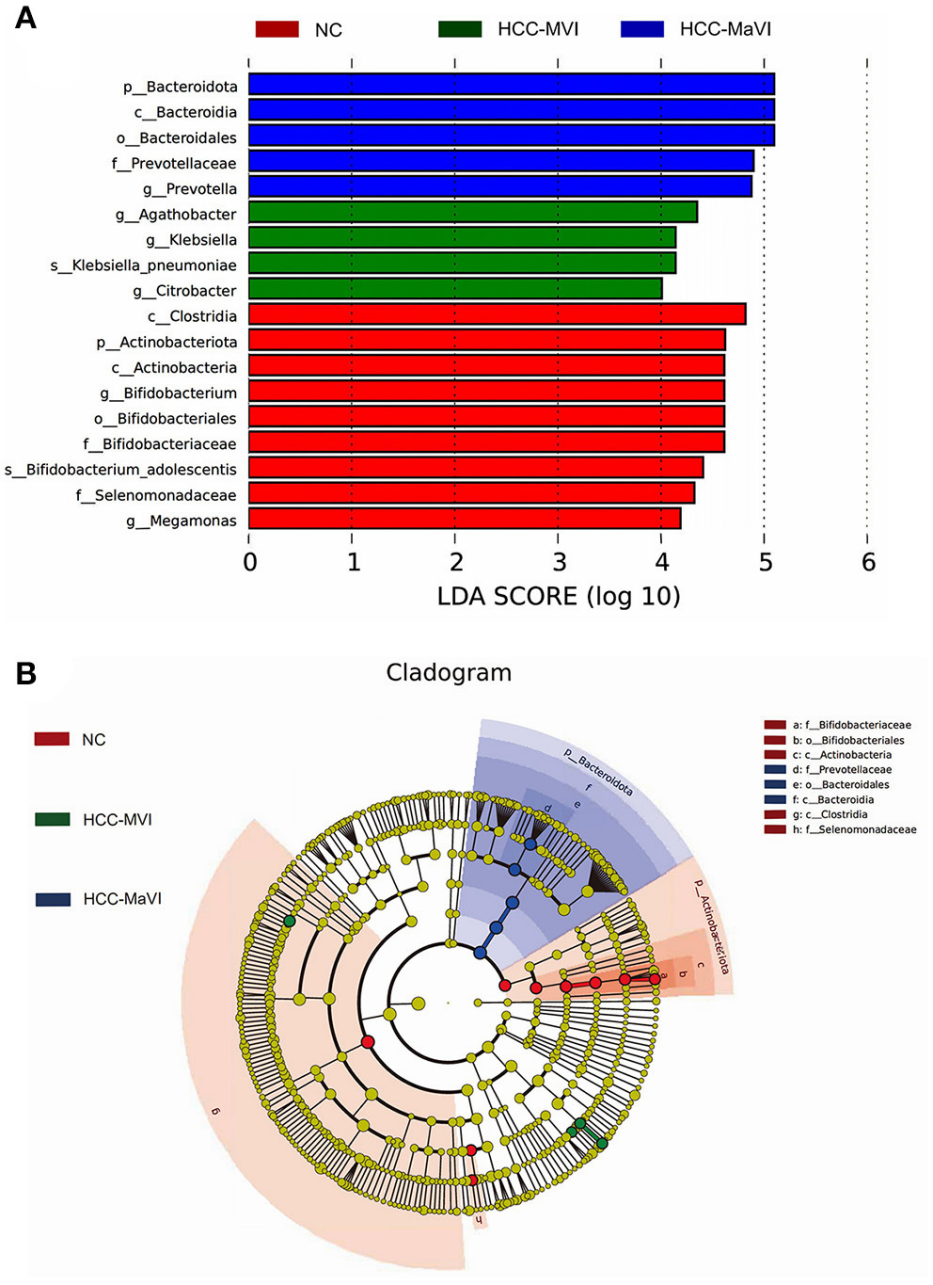
LEfSe analysis of gut microbiota. **(A)** LefSe analysis showed significant bacterial differences among NC, HCC-MVI, and HCC-MaVI groups; **(B)** A cladogram of different taxonomic compositions among NC, HCC-MVI, and HCC-MaVI groups.

In addition to diversity, the LEfSe analysis was applied to identify microbiome signatures that were differentially enriched among the NC, HCC-MVI, and HCC-MaVI groups. The result showed that genus*_Agathobacter*, genus *Citrobacter*, genus*_Klebsiella*, and species*_Klebsiella*_*pneumoniae* were significantly enriched in HCC-MVI samples. Accordingly, several microbes, such as *Prevotellaceae, Bacteroidia, Prevotella, Bacteroidales*, and *Bacteroidota* exhibit enrichment in patients with HCC-MaVI (*p* < 0.05, LDA score > 4; Figures 5A,B).

### The Potential Microbial Model Established for HCC-MVI Prediction

**Figure 6:**
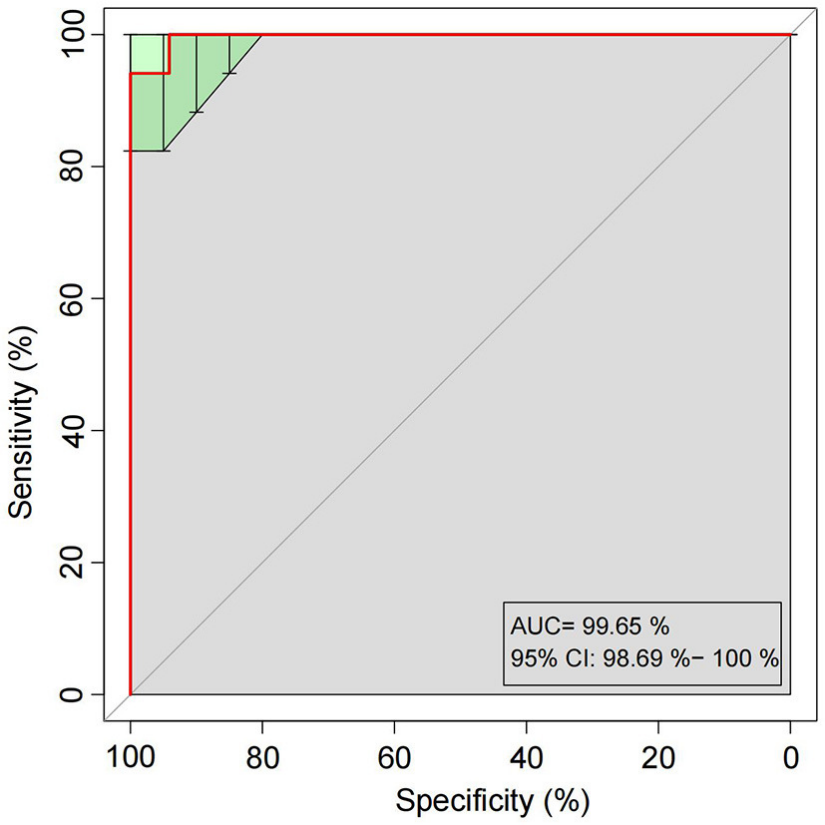
Receiver operating characteristic (ROC) curves built with twenty bacterial families for HCC-MVI prediction.

A random forest model was applied to predict HCC-MVI using 20 bacterial families (including *Clostridiaceae, UCG_010, Tannerellaceae, Ruminococcaceae, Eubacterium coprostanoligenes group, Synergistaceae, Monoglobaceae, Rikenellaceae, unidentified Gastranaerophilales, Rhodobacteraceae, Dysgonomonadaceae, Bifidobacteriaceae, Burkholderiaceae, Enterobacteriaceae, Morganellaceae, Atopobiaceae, Marinifilaceae, Erysipelatoclostridiaceae, Bacteroidaceae, and Desulfovibrionaceae*) with a significant difference among groups, which was filtered out based on mean decrease accuracy and mean decrease gin analysis. The receiver operating characteristic (ROC) analysis was performed to validate the prediction accuracy of this potential model for HCC-MVI with the calculated area under the curve (AUC) being 94.81% (95% CI: 87.63–100%), illustrating a good predictive performance of this model (Figure 6 and Supplementary Figure 11).

### Gut Microbiota Associated With TME Changes in HCC-MVI Patients

**Figure 7:**
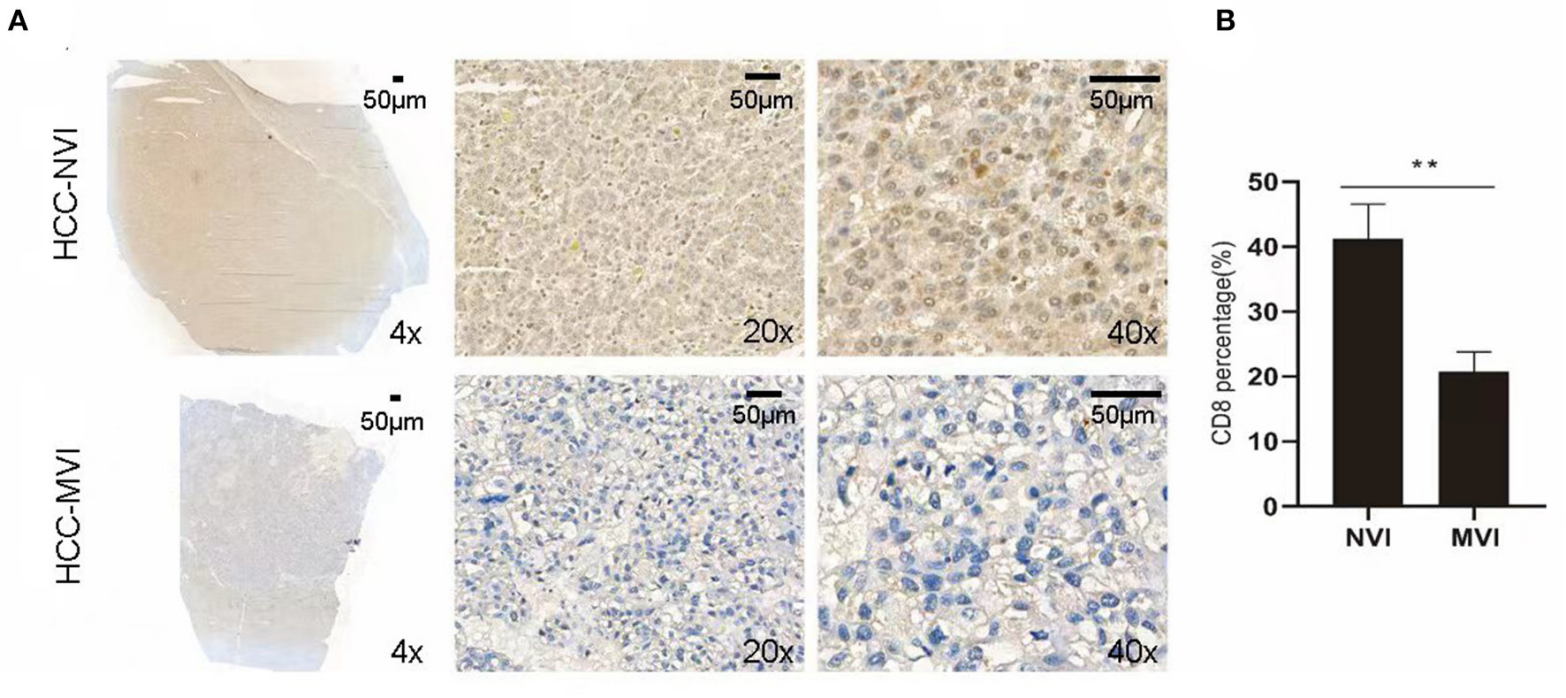
Immunohistochemical profiles of HCC. **(A)** Representative immunohistochemistry images reveal increased expression of CD8^+^ T cell in the HCC-MVI and HCC-NVI group (4 × , 20 × and 40 × magnification, respectively). Scale bar: 50 μm. **(B)** Percentage of CD8^+^ T cell in HCC-MVI and HCC-NVI groups, **P < 0.01 by t-test.

Intratumoral interactions between the cellular and structural components of the tumor microenvironment (TME) can regulate cancer cell survival, local invasion, and metastatic dissemination (27). Immunohistochemical staining was thus applied to analyze the positive rate of CD8^+^ T cells in the TME group of liver cancer with or without MVI. Compared with the HCC-NVI group, the positive rate of CD8^+^ T cells in the HCC-MVI TME was significantly decreased (41.3 vs. 20.8% *P* = 0.001; Figures 7A,B).

**Figure 8:**
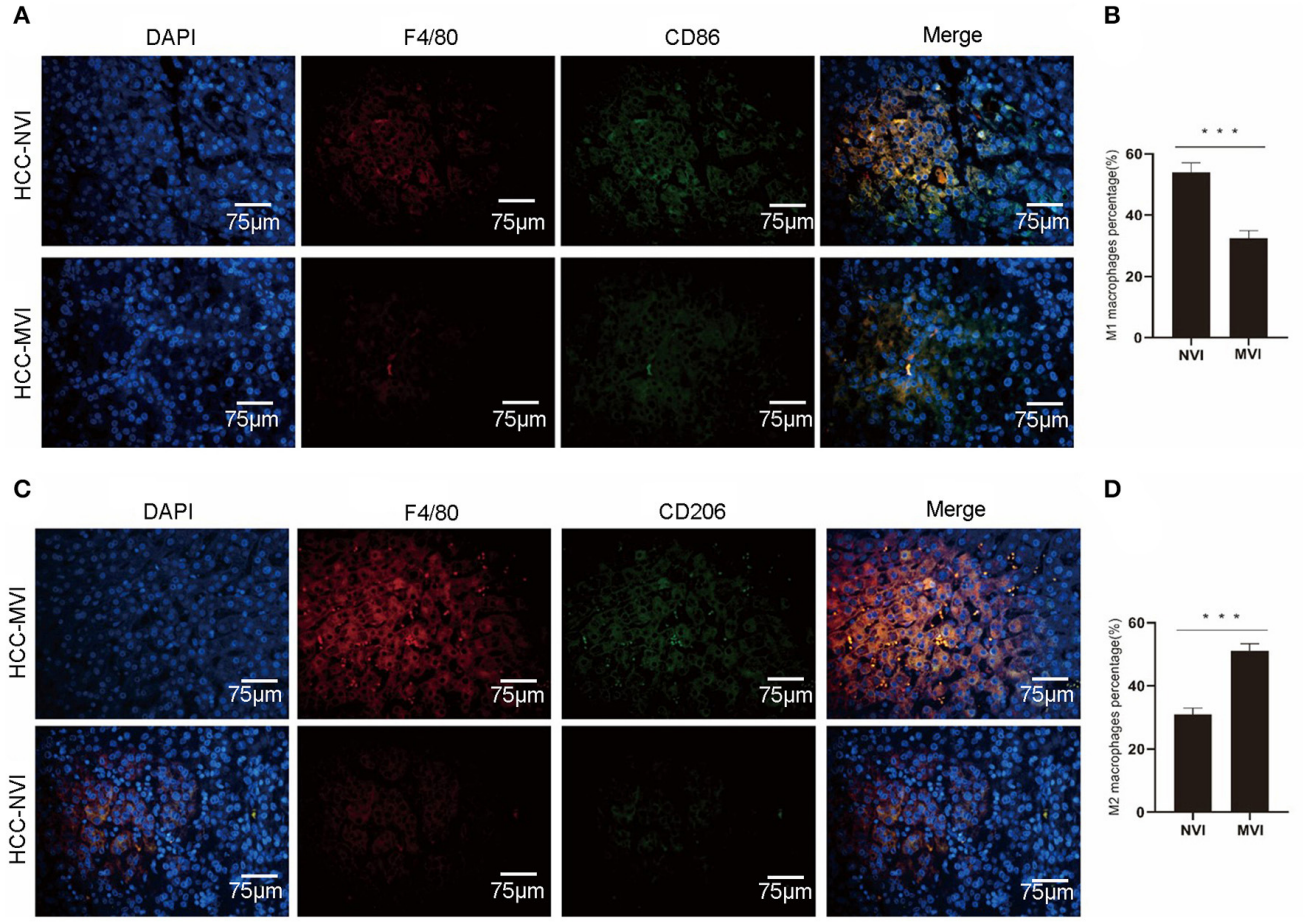
Immunofluorescence profiles of HCC. Representative image **(A)** and calculation **(B)** of M1 TAM positive rate in the HCC-MVI and HCC-NVI groups (40 × magnification). Representative image **(C)** and calculation **(D)** of the M2-type TAM positive rate in the HCC-MVI and HCC-NVI groups (40 × magnification). Scale bar: 75 μm, ***P < 0.001 by t-test.

An immunofluorescence study was applied to further analyze M1 and M2 tumor-associated macrophage (TAM) cells in the TME of patients with HCC. The results indicate that compared with the HCC-NVI group, the positive rate of M1 TAM in the HCC-MVI group was significantly lower (32.5 vs. 54.0%, *P* < 0.001), whereas the positive rate of M2-type TAM cells in the HCC-MVI group was significantly increased (51.1 vs. 31.0%, *P* < 0.001; Figure 8).

### Correlation Analysis Between the Intestinal Microbiome and TME Indicators

**Figure 9:**
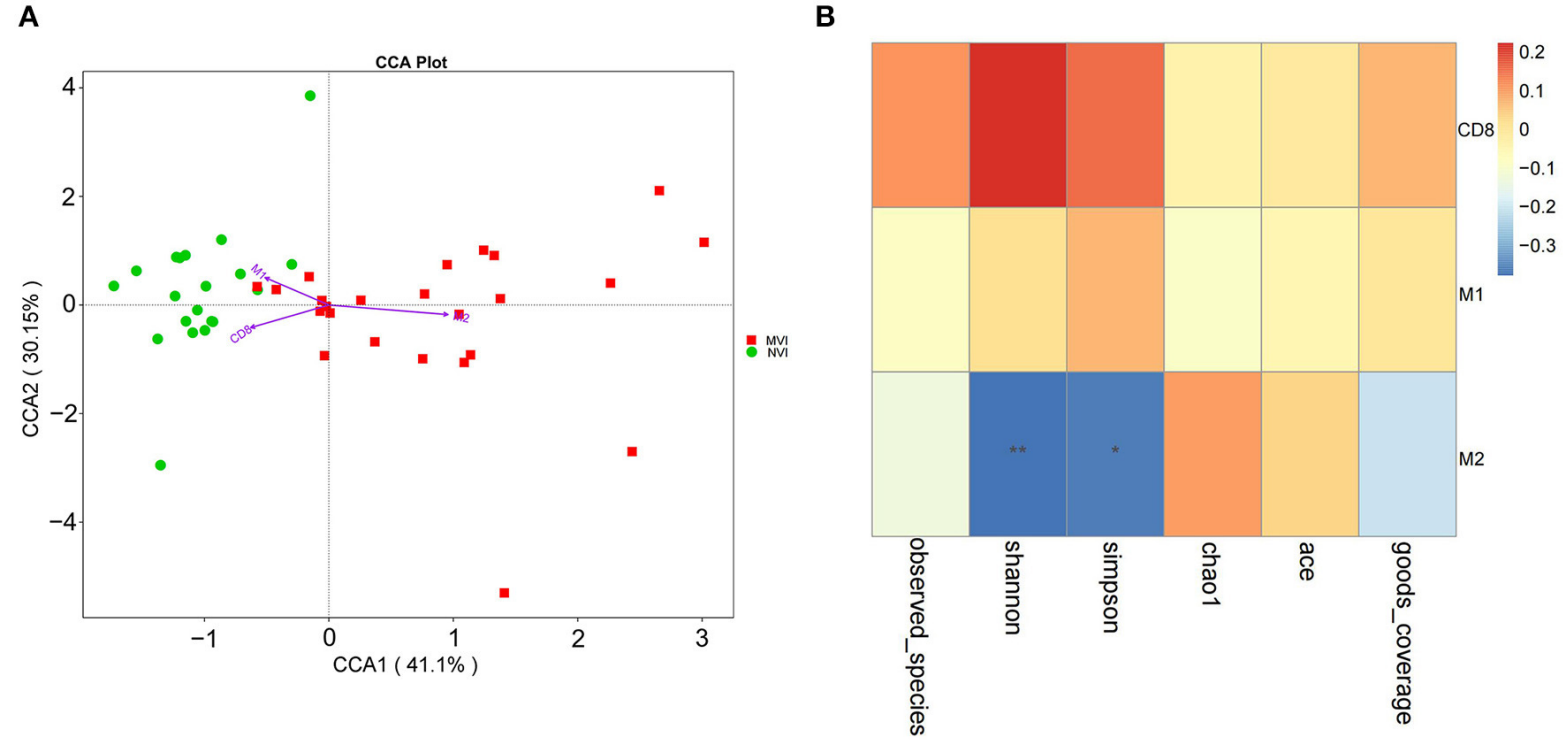
Correlation analysis of TME indicators and intestinal flora. **(A)** CCA analysis of intestinal flora and CD8^+^ T cells, M1 and M2-type TAM cells in HCC-MVI and HCC-NVI groups; **(B)** Spearman correlation analysis of CD8^+^ T cells, M1 and M2 type TAM cells in HCC-MVI group.

Cumulative contribution analysis (CCA) is used to explore the correlation among the positive rate of CD8^+^ T cells, M1- and M2-type TAM cells in TME, and the intestinal microbiome of different vascular invasions. As shown in Figure 9A, the cumulative contribution of TME indicators is 37.29% for CCA1 and 22.17% for CCA2, and the results indicate that there is a significant correlation between M2-type TAM and HCC-MVI group intestinal microbiome.

Based on Spearman correlation analysis, correlation among CD8^+^ T cells, M1/M2-type TAM cells, and HCC-MVI group intestinal microbiome was explored in TME. The positive rate of M2-type TAM cells is negatively correlated with the alpha diversity of the HCC-MVI intestinal microbiome (*P* < 0.01, Figure 9B). The positive rate of M2-type TAM in its TME increases once the diversity of the HCC-MVI intestinal microbiome decreases or the microbiome becomes disordered (dysbiosis).

### Functional Analysis of HCC–MVI Microbiome and Its Correlation With M2-type TAM

Based on Tax4Fun, we further explored the functional alterations of the microbiome in the HCC-MVI and HCC-NVI groups (Supplementary Figure 12). The function of the HCC-MVI intestinal microbiome was significantly concentrated in pathways, including replication and repair, translation, energy metabolism, nucleotide metabolism, glycan biosynthesis, and metabolism, while the intestinal microbiome function of HCC-NVI patients was significantly enriched in pathways such as membrane transport, signal transduction, and cell motility (all *p* < 0.05). Based on the KEGG database, we further explored the correlation between the functional pathway of HCC-MVI microbiome and the positive rate of M2-type TAM in TME, and the mTOR signaling pathway was found to be positively correlated with a positive rate of M2-type TAM in TME (correlation coefficient 0.37, *P* = 0.01; Table 2). Taken together, these results suggest that the increase of TAM M2 positive rate in HCC-MVI TME may be involved in the mTOR signaling pathway.

**Table 2:** The correlation between the functional pathway of microbiome and the positive rate of M2 type TAM in HCC-MVI group based on the KEGG database.

**Signaling pathway**	**r**	***P-value***
Adipocytokine signaling pathway	0.17	0.27
AMPK signaling pathway	0.02	0.89
Calcium signaling pathway	0.065	0.66
cAMP signaling pathway	0.0061	0.97
HIF-1 signaling pathway	−0.15	0.32
IL-17 signaling pathway	0.088	0.55
**mTOR signaling pathway**	**0.37**	**0.01**
NF-kB signaling pathway	−0.071	0.64
NOD-like receptor signaling pathway	0.025	0.87
p53 signaling pathway	0.079	0.6
Phosphatidylinositol signaling system	−0.0093	0.95
PI3K/Akt signaling pathway	0.055	0.71
PPAR signaling pathway	0.11	0.47
Ras signaling pathway	0.073	0.63
Sphingolipid signaling pathway	0.075	0.62
TNF signaling pathway	−0.071	0.64
VEGF signaling pathway	−0.071	0.64

## Discussion

In this study, we comprehensively explored the effects of the fecal microbiome on vascular invasion in patients with HCC, correlating them with clinical data and assessing whether a microbiome study can gain insight into a potential HCC-MVI prediction model. We showed that HCC-MVI significantly affects the relative abundance of several gut bacteria and that a microbial prediction model (based on 20 significant signature microbes) has the potential for predicting and discriminating high-risk patients with HCC-MVI. We also demonstrated that the characteristics of the fecal microbiome in patients with HCC may be associated with TME.

Several studies have shown that gut microbiota plays a crucial role in tumor prognosis and response to immunotherapy/chemotherapy (28). However, the effects of gut microbiota on MVI are largely unknown. As an important prognosis factor of HCC, current MVI assessment mainly depends on pathology following surgery. Therefore, preoperative prediction of MVI, especially in a non-invasive manner, is of great significance. Several studies have evaluated the performance of MVI predictive models constructed with clinical and molecular indicators, and the AUC of these models ranged from 0.73 to 0.81, suggesting that higher specificity and sensitivity are crucial for future studies (29–31). Intestinal microbiome analysis becomes an important direction for predictive model construction considering its convenience and non-invasive sample collecting requirements. Previously, Zheng et al. (32) screened out six important microbial markers at the genus level among hepatitis B virus (HBV)-related patients with HCC and healthy controls. They constructed predict models to explore the predictive value of intestinal microbiome on posthepatectomy recurrence and survival. The AUC of 2-year RFS survival predicted value was 68%, and the 5-year OS predicted AUC was 81%. Currently, there is no research that can predict HCC-MVI based on intestinal microbiome analysis.

In the present study, we performed comprehensive fecal microbiome analysis among the healthy controls (NC) and patients with HCC with different vascular invasion statuses (the HCC-NVI, HCC-MVI, and HCC-MaVI groups). The major microbiome component of healthy controls is found to be *Firmicutes* (with the percentage of 50–80%), followed by *Bacteroides, Proteobacteria, Actinomycetes, and Verrucomicrobia*. Our research indicated that the abundance of *Bacteroides* significantly enriched in patients with HCC while *Firmicutes* were reduced, which is consistent with previous studies (32–34). Compared with the HCC-NVI group and NC group, the abundances of *Prevotella* and *Klebsiella* were significantly increased in the HCC-MVI group. *Prevotella* was enriched in hepatitis C virus-related HCC and regarded as an independent prognostic biomarker in ESCC patients (30, 35). *Prevotella's* lipopolysaccharides (LPS) have a proinflammatory effect and its abundance *is* negatively correlated with interleukin (IL)-17A in colorectal cancer samples while the correlation with IL-9 is positive (36). *Klebsiella* has been shown to have increased abundance in patients with a variety of cancers including pancreatic cancer, colon cancer, and esophageal cancer (37–39). It was also related to the lymphatic metastasis and prognosis of pancreatic cancer (38). *Klebsiella pneumoniae* may promote chemoresistance to adjuvant gemcitabine treatment while following intervention with antibiotic quinolone can improve survival (40). Interestingly, our results indicated that *Monoglobales*, a member of microbiota which has not been reported in liver disease or cancer study, shows gradually decreased abundancy in the four subgroups according to differential vascular invasion status (highest in NC, then in HCC-NVI, HCC-MVI, and lowest in HCC-MaVI). Therefore, it may serve as a potential biomarker for negatively predicting HCC vascular invasion. However, with a limited study reported and finite evidence, the role of gut bacteria *Monoglobales* in HCC-MVI needs to be further explored.

As an emerging investigative field, microbiome study has some advantages over clinical or molecular indicators, especially when patients prefer non-invasive and convenient sample collection procedures. Wang et al. conducted a nomogram that identified four risk factors (tumor size, number of tumors, neutrophils, and serum α-fetoprotein) for MVI prediction (29). Circulating tumor DNA (ctDNA) was also identified in operable patients with HCC, suggesting that ctDNA may function as an independent risk factor for MVI and facilitate precise treatment strategies (41). Compared with aforesaid MVI prediction methods, the microbial model we constructed for HCC-MVI prediction obtained better performance with higher AUC (94.81%). The advent of high throughput next-generation sequencing (NGS) technologies has revolutionized microbiome study, especially when combined with advances in bioinformatics analysis and machine learning. Thus, the gut microbiome should be considered as a viable diagnostic and therapeutic target, which can be applied for assessing the clinical impact of these findings in future studies.

Currently, the mechanisms underlying the effects of microbiota on tumor prognosis remain unclear. Several clinical and subcutaneous preclinical studies revealed that gut microbiota was associated with systemic inflammation, including inflammatory responses in tumors (42–46). Our study revealed that the M2-type TAM positive rate was significantly higher in the HCC-MVI group than that of the HCC-NVI group. Moreover, once the diversity of the HCC-MVI microbiome decreases and the intestinal microbiome becomes disordered, the M2-type TAM positive rate in TME shows a further increase. These results are in accordance with previous studies which have shown that high expression of M2-type TAM in tumor tissue can promote tumor invasion and metastasis, resulting in poor prognosis (24, 47). The correlation between functional pathways and TME M2-type TAM was further explored, and the results suggest that the increase of M2-type TAM positive rate may be involved in the mammalian target of rapamycin (mTOR) pathway. The mTOR is frequently deregulated in tumorigenesis, activating somatic mutations of mTOR, which were recently identified in several types of cancer, and hence mTOR is therapeutically targeted (48).

There are certain limitations in the present study. First, some of the results obtained were achieved based on limited sample size and/or lack of further validation, therefore future validation in larger cohorts may reduce the false positive value and unreliability during HCC-MVI screening. Although the M2-type TAM and mTOR metabolic pathways are initially predicted to be involved based on the microbiome data, the related mechanism of their interaction needs to be further explored. However, the fact that these correlations were evident even in this limited sample size testifies to the potentialities of the proposed model. Interpreting changes was not the aim of our current study, thus we did not assess other clinical parameters such as intestinal permeability, inflammatory status, dietary habits, comorbidities, comedications, and portal hypertension, resulting in insufficient functional understanding of observed microbial changes. Moreover, the present study focused on the differences of gut microbiota resulting from MVI among HBV-related patients with HCC. Involving benign controls with HBV infection in subsequent larger cohort studies may help us refine the prediction model with less variables and higher AUC. Finally, the concomitant fecal metabolomic analysis would be a valuable addition to microbiome data for a more comprehensive understanding, especially on how the observed microbiota changes during MVI development.

However, strengths also are worthy of mention. As far as we know based on a literature search, this is the first clinical study exploring the influence of the gut microbiome on HCC-MVI development, and, as such, its results should be regarded with attention. In particular, we generated the first prediction model for HCC-MVI based on microbiome correlative analysis, which obtains high prediction performance. The microbial model already showed discriminative properties under the current experimental condition. It is likely that expanding the research among a larger cohort as well as other clinical parameters as mentioned above will further disclose the diagnostic potential of this method. Based on the microbiome data, the metabolic pathways that may be involved are initially predicted; however, the mechanism of their interaction needs to be further explored.

In conclusion, the present study highlights that gut microbiome alterations may have an influence on HCC-MVI prediction and relate to M2-type TAM of TME. Moreover, it shows that the microbial prediction model is able to efficiently predict HCC–MVI development. These preliminary results need to be confirmed and expanded in larger HCC cohorts and other cancer populations. The animal study is also needed to understand whether the differential microbiome affects the development of HCC-MVI by exploring possible mechanisms or the other way around. If confirmed, they might open the way to a quick and low-cost non-invasive diagnosis with many potential perspectives not only in hepatic vascular invasion diseases. Therefore, this clinical study provides a strong potential of translational implications from bench to bedside helping the clinicians in terms of disease prediction and diagnosis.

## Data Availability Statement

The data presented in the study are deposited in the China National Microbiology Data Center, accession number NMDCX0000120 (https://nmdc.cn/resource/attachment/detail/NMDCX0000120).

## Ethics Statement

The studies involving human participants were reviewed and approved by Tianjin Medical University Cancer Institute and Hospital. The patients/participants provided their written informed consent to participate in this study.

## Author Contributions

WLu, EE-O, XZ, and NZ conceived the study. NZ, ZW, JL, and SZ recruited participants. ZW, JL, SW, and YL conducted the analysis. TL and WLi analyzed fecal samples. ZW, LG, and YL wrote the article. All authors revised and approved the written manuscript.

## Funding

This research was supported by the Tianjin Science and Technology Program (No. 19YFZCSY00020).

## Conflict of Interest

The authors declare that the research was conducted in the absence of any commercial or financial relationships that could be construed as a potential conflict of interest.

## Publisher's Note

All claims expressed in this article are solely those of the authors and do not necessarily represent those of their affiliated organizations, or those of the publisher, the editors and the reviewers. Any product that may be evaluated in this article, or claim that may be made by its manufacturer, is not guaranteed or endorsed by the publisher.
