# Supplementary material for: Characterization of Gut Microbiota and Exploration of Potential Predictive Model for Hepatocellular Carcinoma Microvascular Invasion
Source: Front Med (Lausanne). 2022 Mar 17;9:836369. doi: 10.3389/fmed.2022.836369 (PMC8971959; doi:10.3389/fmed.2022.836369)
Supplement: Supplementary file 1 [file Data_Sheet_1.docx]

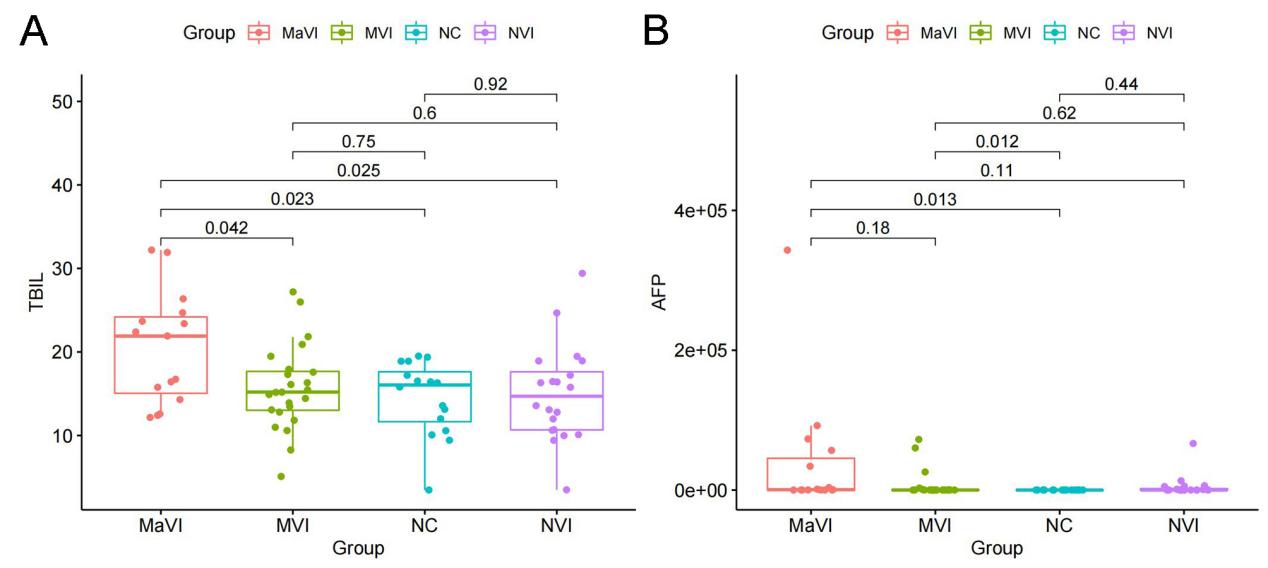
Supplementary Material

**Supplementary Figure 1.** Differential levels of TBIL (A) and AFP (B) in the NC group and the HCC groups (MaVI, MVI, NVI).


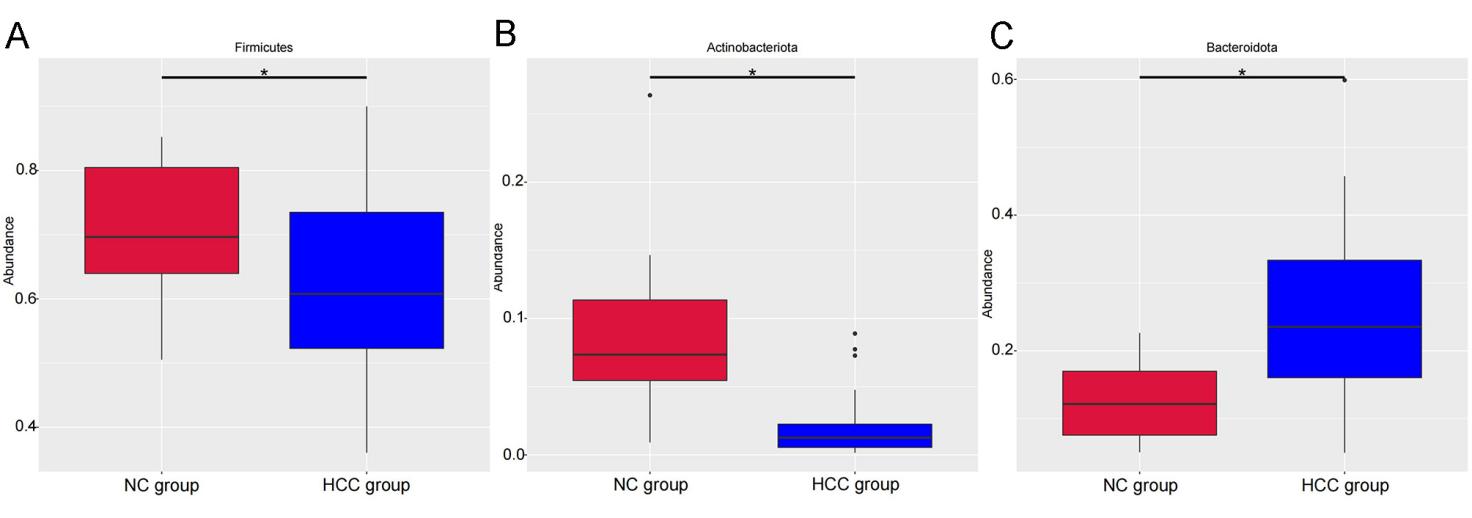


**Supplementary Figure 2.** Differential abundance of gut microbes at the phylum level. Abundance of Firmicutes (A), Actinobacteriota (B), Bacteroidota (C) in the NC group and the HCC group.


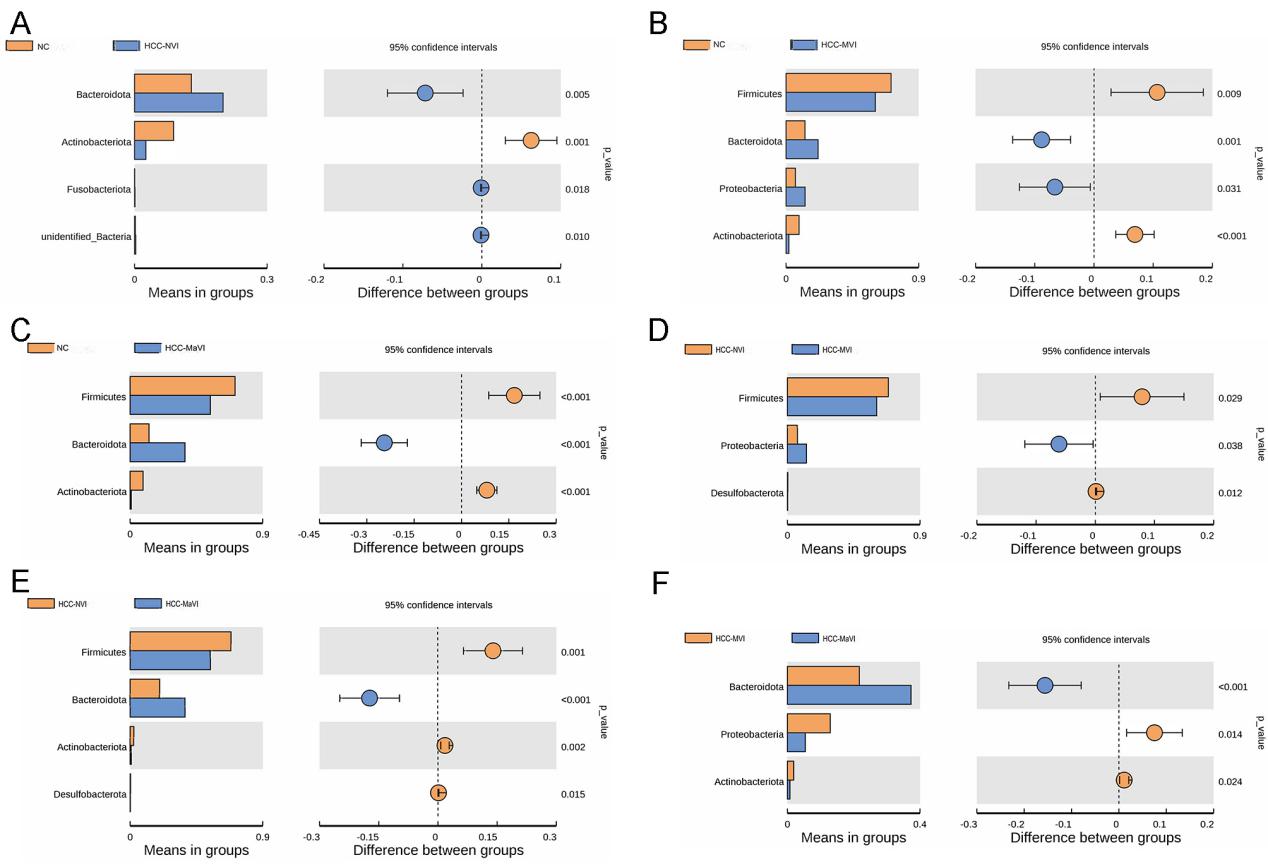


**Supplementary Figure 3.** Differential enrichment of gut microbes at the phylum level in the NC, HCC-NVI, HCC-MVI and HCC-MaVI groups. Differential abundance in the NC and HCC-NVI groups (A), in the NC and HCC-MVI groups (B), in the NC and HCC-MaVI groups (C), in the HCC-NVI and HCC-MVI groups (D), in the HCC-NVI and HCC-MaVI groups (E), in the HCC-MVI and HCC-MaVI groups (F).


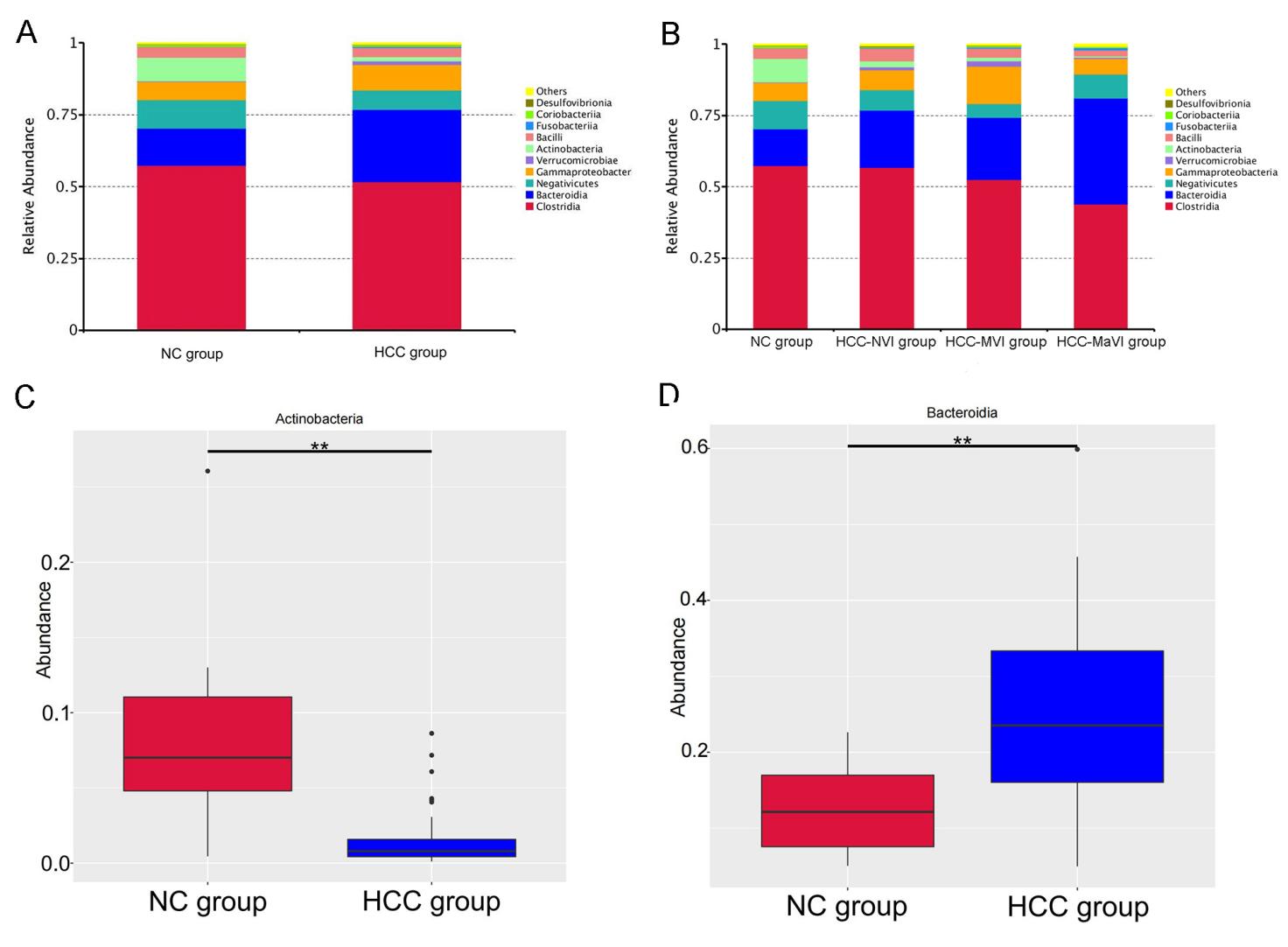


**Supplementary Figure 4.** Differential abundance of gut microbes at the class level. (A) Top ten abundant gut microbes in the HCC and NC groups. (B) Top ten abundant microbes in the NC, HCC-NVI, HCC-MVI and HCC-MaVI groups. Abundance of Actinobacteria (C) and Bacteroidia (D).


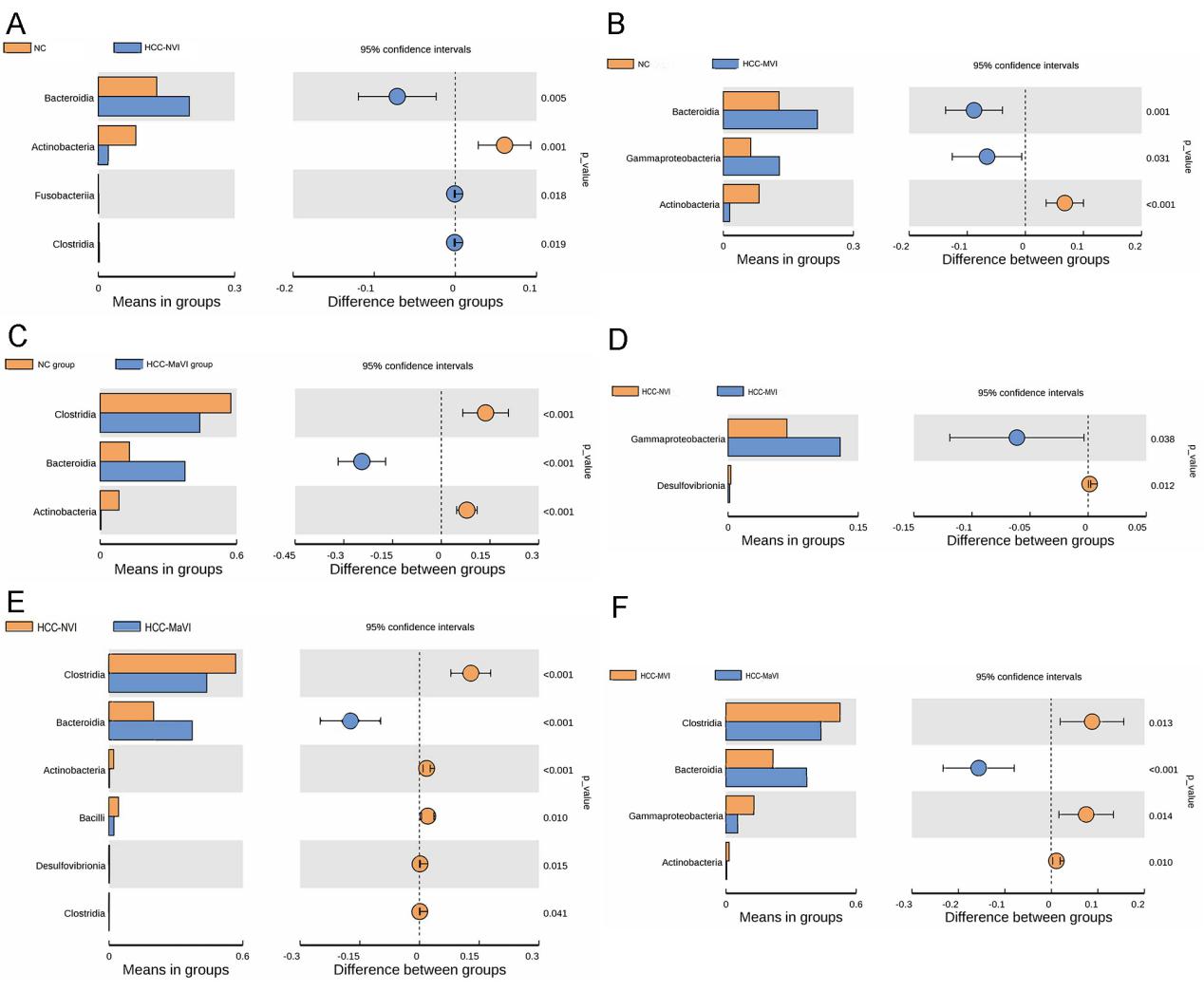


**Supplementary Figure 5.** Differential enrichment of gut microbes at the class level in the NC, HCC-NVI, HCC-MVI and HCC-MaVI groups. Differential abundance in the NC and HCC-NVI groups (A), in the NC and HCC-MVI groups (B), in the NC and HCC-MaVI groups (C), in the HCC-NVI and HCC-MVI groups (D), in the HCC-NVI and HCC-MaVI groups (E), in the HCC-MVI and HCC-MaVI groups (F).


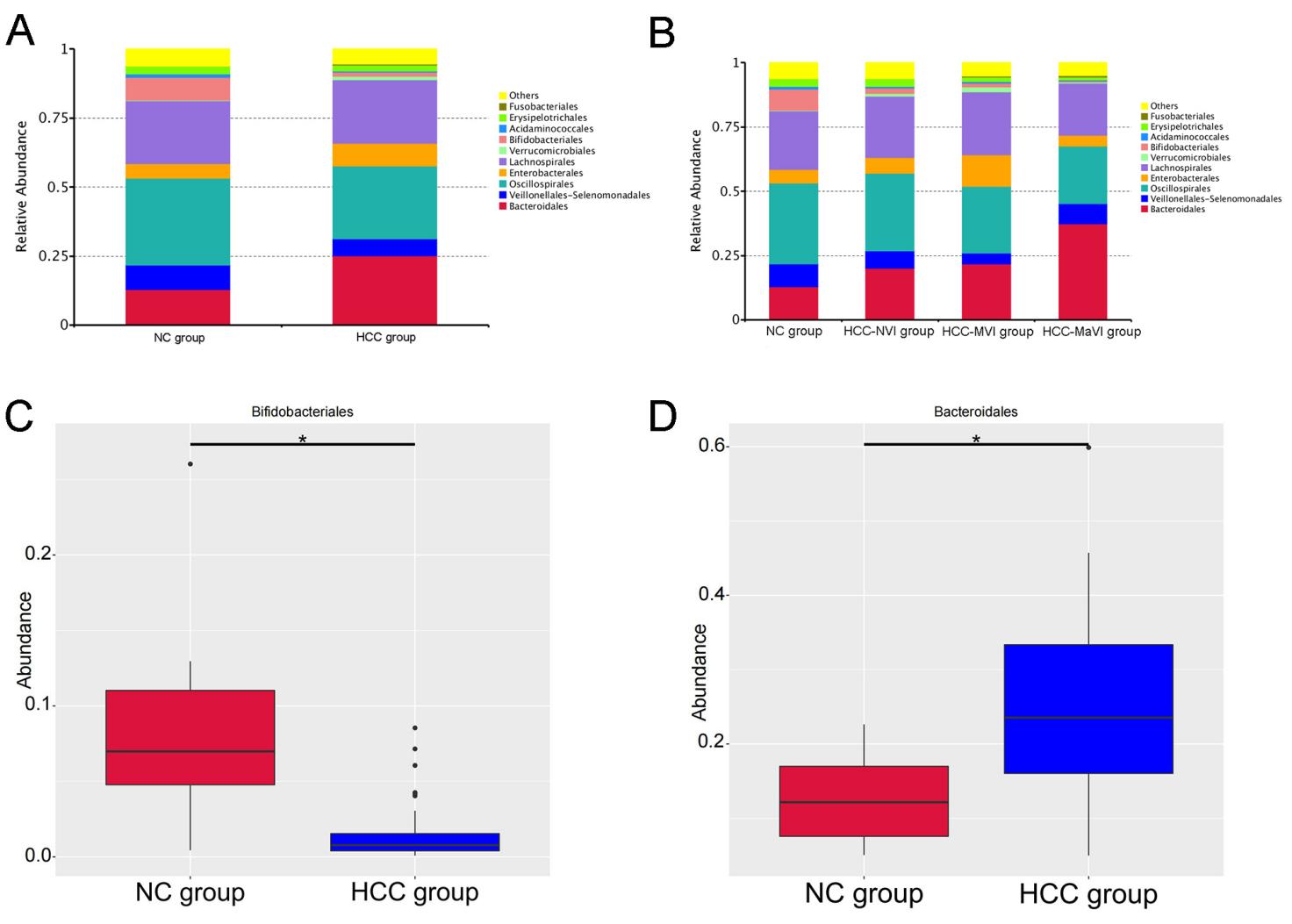


**Supplementary Figure 6.** Differential abundance of microbes at the order level. (A). Top ten abundant microbes in the HCC and NC groups. (B). Top ten abundant microbes in the NC, HCC-NVI, HCC-MVI and HCC-MaVI groups. Abundance of Bifidobacteriales (C) and Bacteroidales (D).


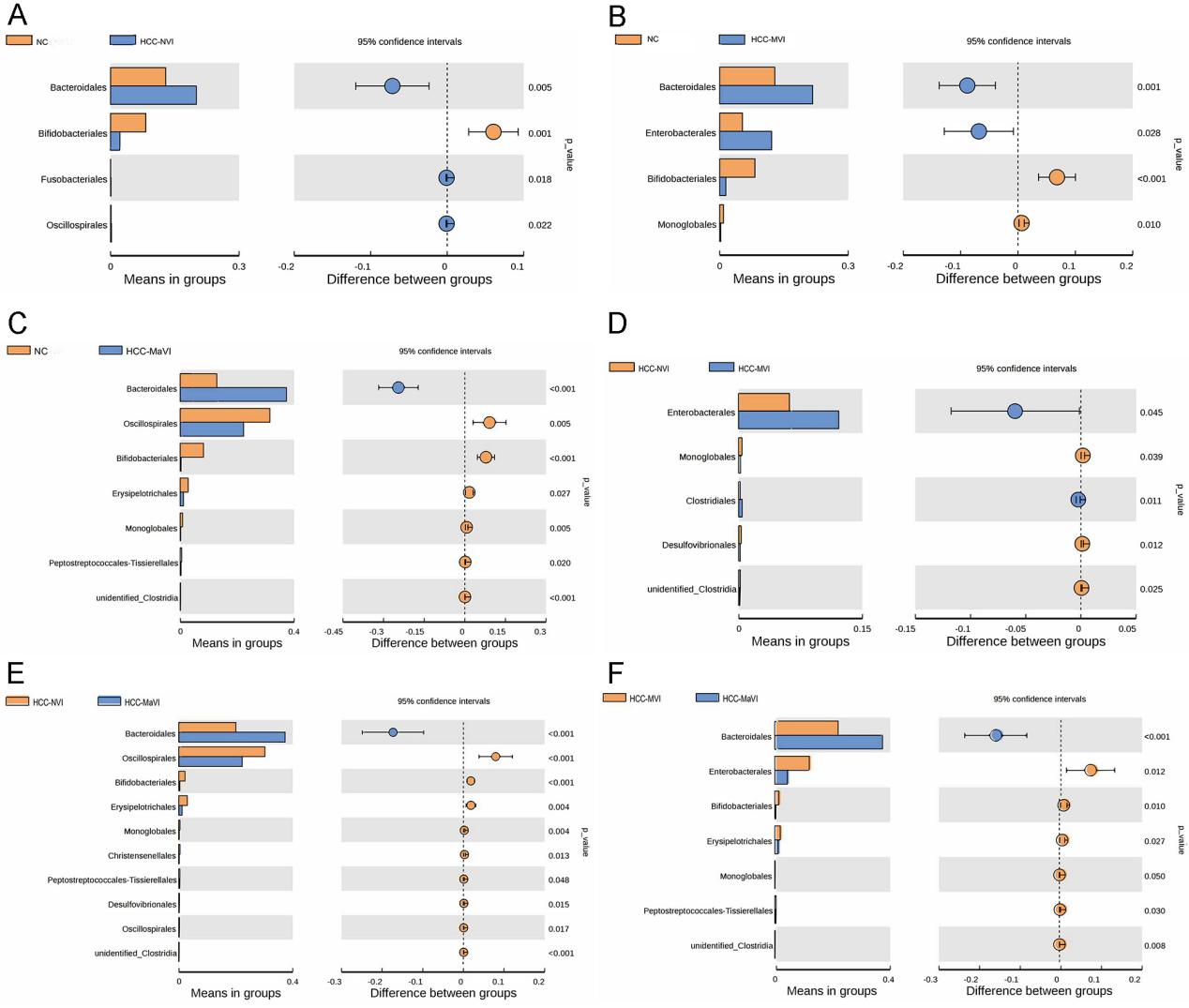


**Supplementary Figure 7.** Differential enrichment of microbes at the order level in the NC, HCC-NVI, HCC-MVI and HCC-MaVI groups. Differential abundance in the NC and HCC-NVI groups (A), in the NC and HCC-MVI groups (B), in the NC and HCC-MaVI groups (C), in the HCC-NVI and HCC-MVI groups (D), in the HCC-NVI and HCC-MaVI groups (E), in the HCC-MVI and HCC-MaVI groups (F).


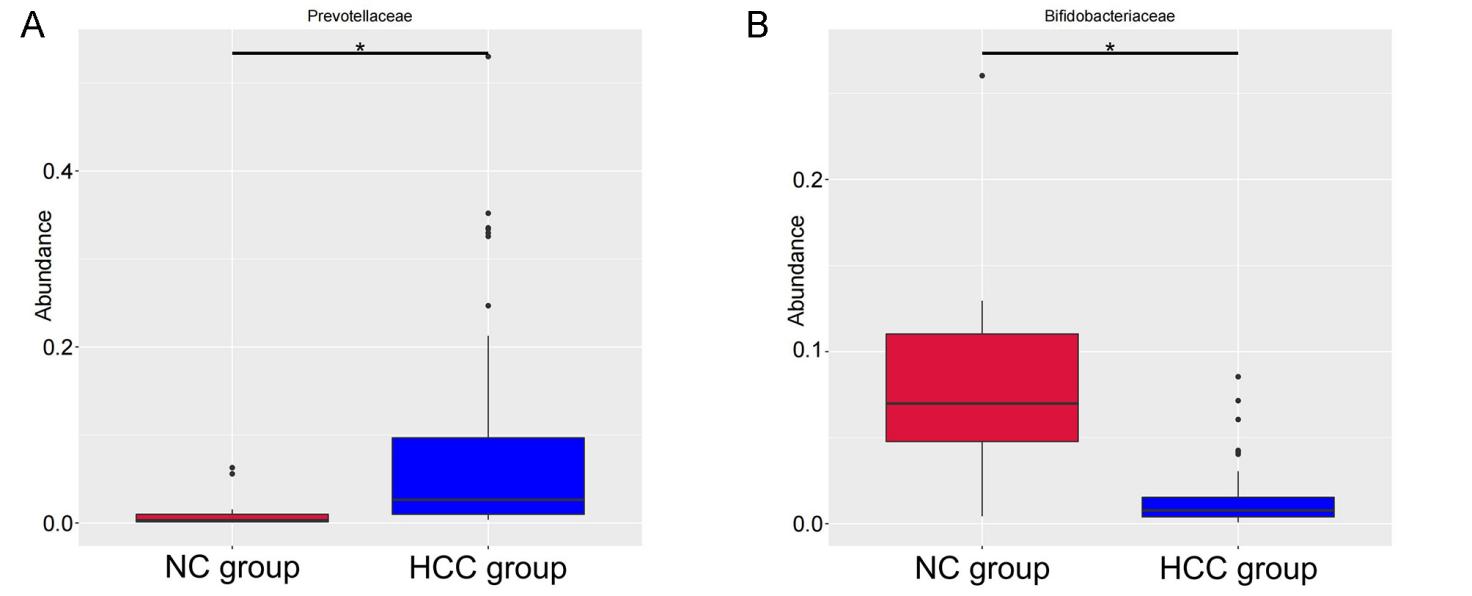


**Supplementary Figure 8.** Differential abundance of microbes at the family level. Abundance of Prevotellaceae (A) and Bifidobacteriaceae (B).


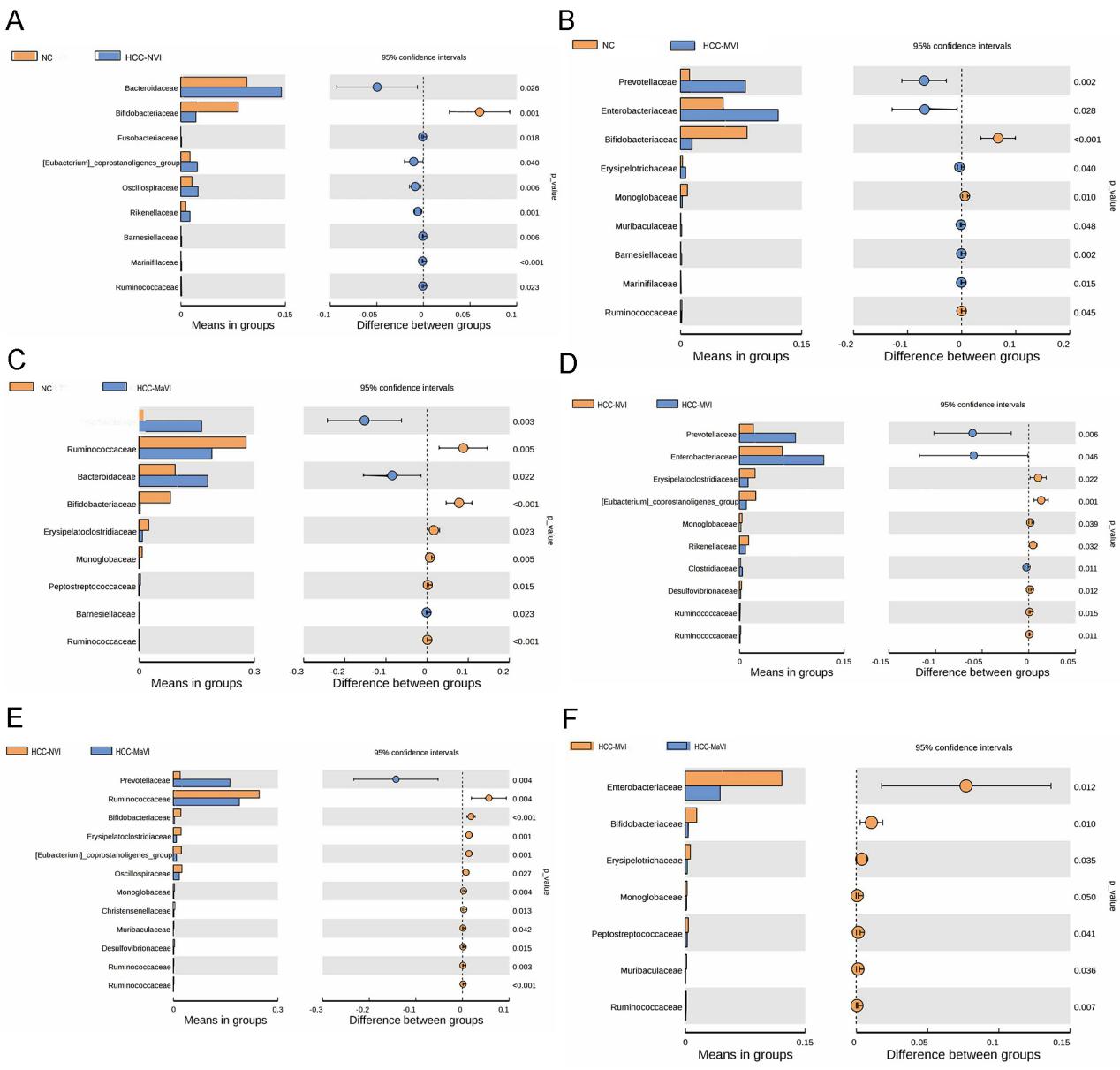


**Supplementary Figure 9.** Differential enrichment of microbes at the family level in the NC, HCC-NVI, HCC-MVI and HCC-MaVI groups. Differential abundance in the NC and HCC-NVI groups (A), in the NC and HCC-MVI groups (B), in the NC and HCC-MaVI groups (C), in the HCC-NVI and HCC-MVI groups (D), in the HCC-NVI and HCC-MaVI groups (E), in the HCC-MVI and HCC-MaVI groups (F).


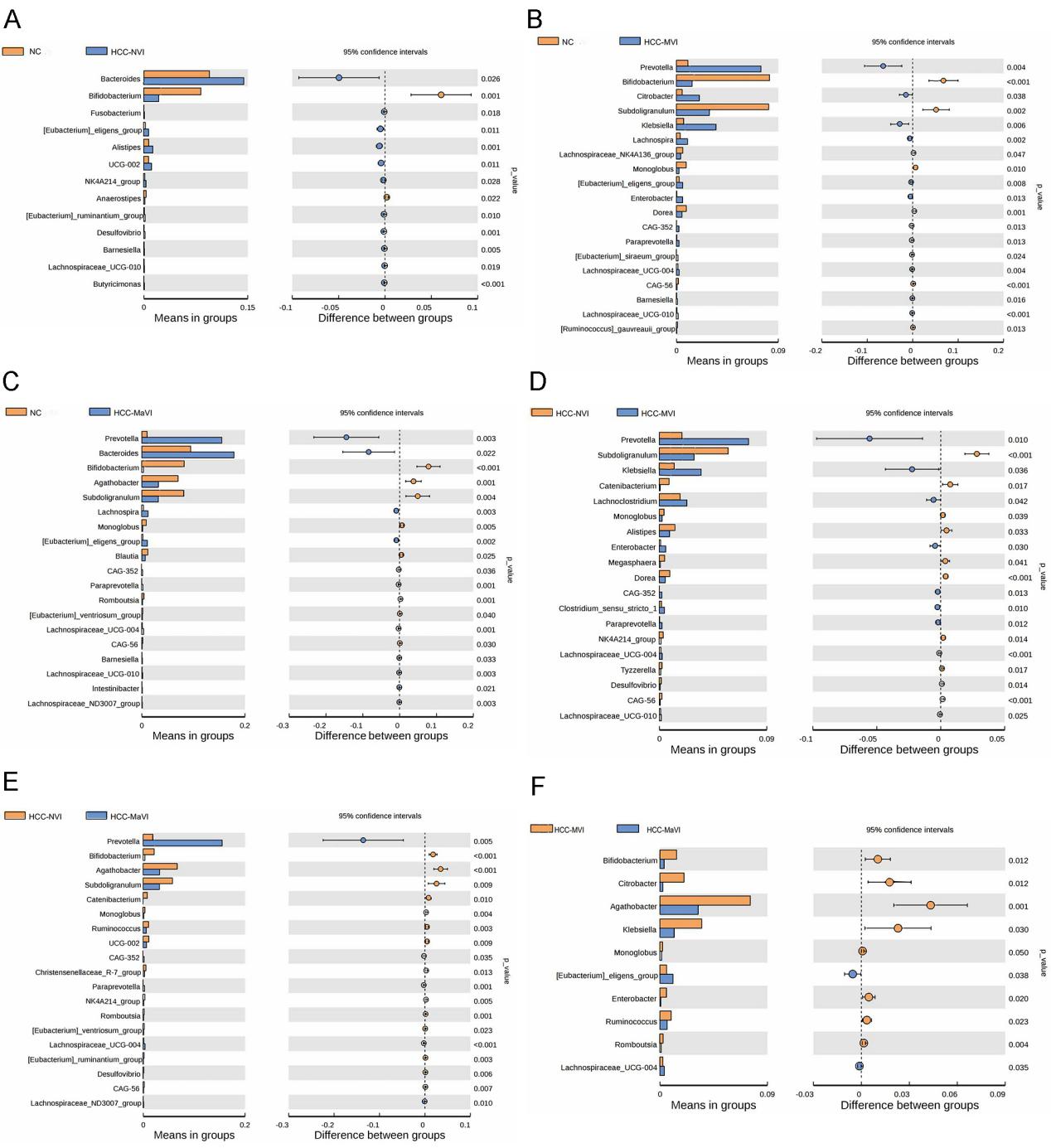


**Supplementary Figure 10.** Differential enrichment of microbes at the genus level in the NC, HCC-NVI, HCC-MVI and HCC-MaVI groups. Differential abundance in the NC and HCC-NVI groups (A), in the NC and HCC-MVI groups (B), in the NC and HCC-MaVI groups (C), in the HCC-NVI and HCC-MVI groups (D), in the HCC-NVI and HCC-MaVI groups (E), in the HCC-MVI and HCC-MaVI groups (F).


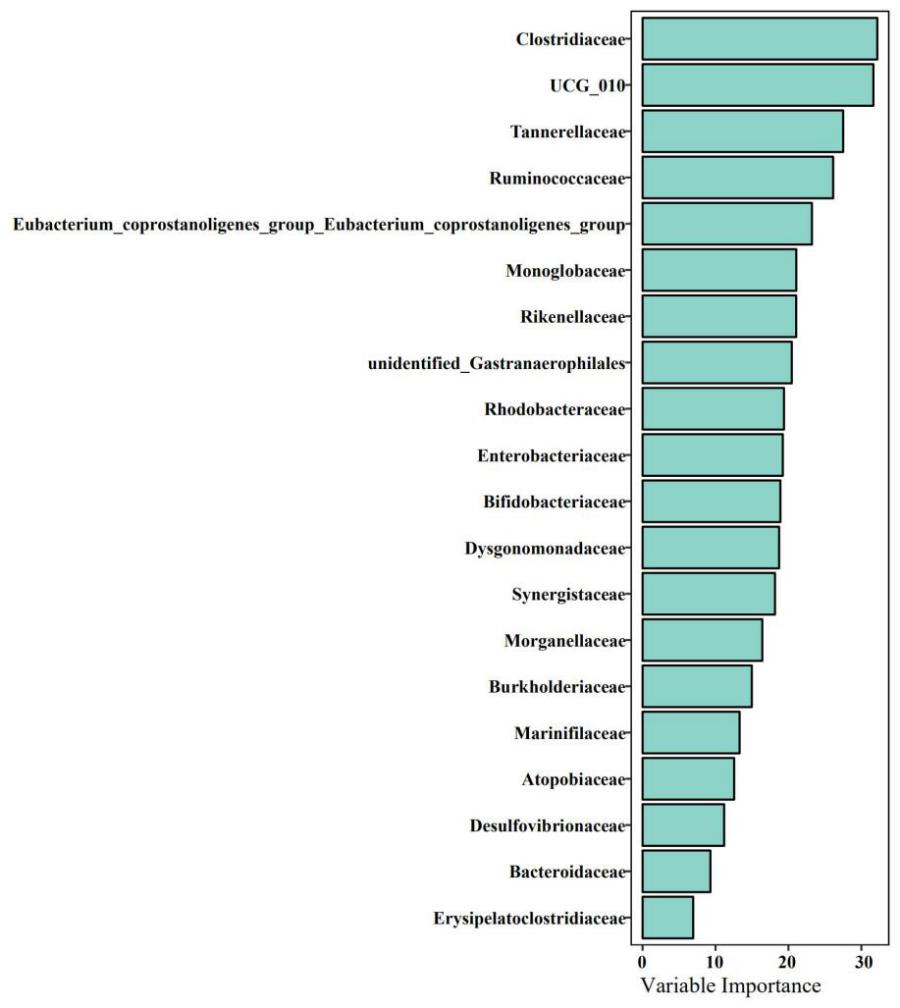


**Supplementary Figure 11**. HCC-MVI microbial prediction model screening variables.


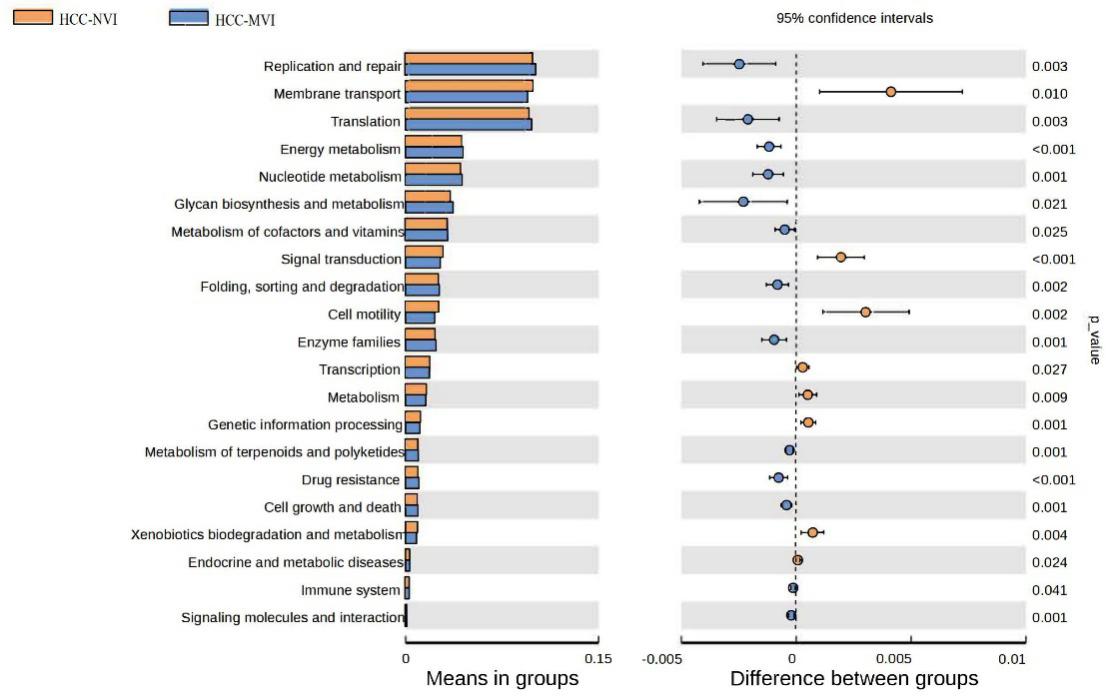


**Supplementary Figure 12.** The functional changes of microbiome in the HCC-MVI and HCC-NVI groups based on Tax4Fun.
